# Brodie’s Abscess Masquerading as Vaso-occlusive Crisis in a Sickle-cell Disease Patient

**DOI:** 10.7759/cureus.7871

**Published:** 2020-04-28

**Authors:** Taha Sheikh, Rawish Fatima, Muhammad Aziz, Mamtha Balla, Claudiu Georgescu

**Affiliations:** 1 Internal Medicine, University of Toledo College of Medicine and Life Sciences, Toledo, USA; 2 Internal Medicine, University of Toledo, Toledo, USA; 3 Internal Medicine, ProMedica Toledo Hospital, Toledo, USA

**Keywords:** sickle cell disease, vaso-occlusive crisis, brodie's abscess, subacute osteomyelitis, osteomyelitis, enterobacter cloacae, chronic osteomyelitis, sickle cell crisis

## Abstract

Brodie’s abscess is a rare form of subacute osteomyelitis, most commonly found in children between the ages of two to fifteen years. It has slight preponderance for males. It is characterised by centrally placed, well-circumscribed abscess within the medulla or metaphysis of long bone, most commonly tibia, surrounded by a sclerotic wall. It is sometimes considered a transitional phase for the development of chronic osteomyelitis due to infection persisting between two to six months without showing any systemic symptoms specific to osteomyelitis. It is assumed that it is clinically quiescent due to its intraosseous location. It rarely presents with overt symptoms, which occurs if either the abscess enlarges to create pressure against the periosteum, or if the purulent material extrudes from the confines from its sclerotic walls. Due to subliminal clinical features and indolent clinical course, radiologic investigations are the diagnostic modality of choice. Diagnosis requires a high degree of suspicion, especially in the scenario of sepsis with an unknown source of infection. We describe a case of Brodie's abscess in a sickle-cell disease patient which presented as episodes of vaso-occlusive crisis repeatedly before it was diagnosed along with a review of the literature.

## Introduction

Osteomyelitis historically has been categorized into acute, subacute, or chronic based on initial onset and presentation of the disease. Subacute osteomyelitis, which is defined as infection lingering between two to six months, usually develops after a previously treated acute case of osteomyelitis. However, it can also present in patients who have not had any previous history of acute infection as well.

Brodie’s abscess is a rare form of subacute osteomyelitis, leading to a centrally-placed, well-circumscribed abscess, surrounded by a sclerotic wall [1]. It is characterized by infection suspected to have persisted between two to six months without showing any systemic symptoms specific to osteomyelitis. Though not a prerequisite, it may be preceded by osteomyelitis or predisposing conditions like trauma, surgery, prosthetics, or immunosuppression. It is often difficult to distinguish between subacute osteomyelitis and chronic osteomyelitis due to the absence of overt symptoms; Brodie’s abscess is even harder to diagnose and a delay is characteristically seen [2]. The incidence of subacute osteomyelitis is approximately 5 in 100,000 children per year in high-income countries but may be higher in low- and middle-income countries [3].

Brodie’s abscess is most commonly seen in the pediatric population. It has a predilection for developing in the metaphysis of long bones. Classically, extremities are more often involved due to the presence of end arteries in the metaphysis of long bones, with the tibia being the most common target followed by the femur [1]. However, other smaller bones lacking a proper metaphysis are also reported to have been involved [4].

Regardless of the site of involvement or severity, *Staphylococcus aureus* has been found to be the most common organism isolated from these lesions, followed closely by Streptococcus species [5].

Patients with sickle-cell disease have increased the propensity of bone involvement with osteomyelitis, with a lifetime prevalence of up to 12%, being only second to the vaso-occlusive crisis. The mechanism is multifactorial and an increased risk of infection is conferred to hyposplenism, impaired complement activity, and the presence of infarcted or necrotic bone. These cycles are predisposed to avascular necrosis, making nidus for infection. Another challenge is the widespread array of pathology with bones in these patients muddles their presentation. Brodie’s abscess is a challenging diagnosis to make due to the paucity of both clinical symptoms and laboratory findings [6]. We report a case of Brodie’s abscess in a sickle-cell disease patient presenting with recurrent vaso-occlusive crisis due to underlying quiescent Brodie’s abscess.

## Case presentation

A 57-year-old African American gentleman with sickle-cell disease, hemoglobin-SS, avascular necrosis of the bilateral hip and right shoulder, with remote right shoulder replacement, presented to the emergency department complaining of pain in multiple large joints. He complained of pain in the hips, knees, shoulders, and right elbow ongoing for three days. It was progressively worsening with no precipitating factors. He denied any fever, chills, nausea, vomiting, chest pain, palpitation, cough but did have mild shortness of breath. 

On presentation, he was afebrile with a heart rate of 104 beats/min, respiratory rate of 24 breaths/min, and blood pressure of 147/90 mmHg. A cardiovascular exam revealed normal heart sounds. A respiratory exam revealed expiratory wheeze but otherwise, no decreased breath sounds or rhonchi were noted. A joint exam showed no tenderness to palpation, warmth, or swelling of any of the joints; however, there was resistance to movement at the hip joints bilaterally due to pain.

Labwork showed white cell count of 24.9 x10^9^/L, hemoglobin 5.5 mg/dL, platelet 390 x10^9^/L, sodium 134, potassium 3.9, blood urea nitrogen (BUN) 31, creatinine 1.7, lactate dehydrogenase (LDH) 344, procalcitonin <0.05, and normal urinalysis. Blood and urine cultures were sent - reticulocyte count >3%, haptoglobin<30 md/dL. Sickle solubility test returned positive indicating active sickling. He was admitted with the diagnosis of vaso-occlusive crisis and a workup for sepsis and the precipitating cause was sought. He received intravenous fluid resuscitation, packed red blood cell (RBC) transfusion, and medication for pain control. However, despite the escalation of the pain control regimen, he was unable to ambulate and had persistent pain in his hips bilaterally. Despite lack of fever and absence of clinical signs of infection (swelling, warmth, tenderness), the persistently elevated white blood cell (WBC) count with worsening right hip pain and weakness, was concerning. Blood, urine, and stool cultures were negative for any growth.

**Figure 1:**
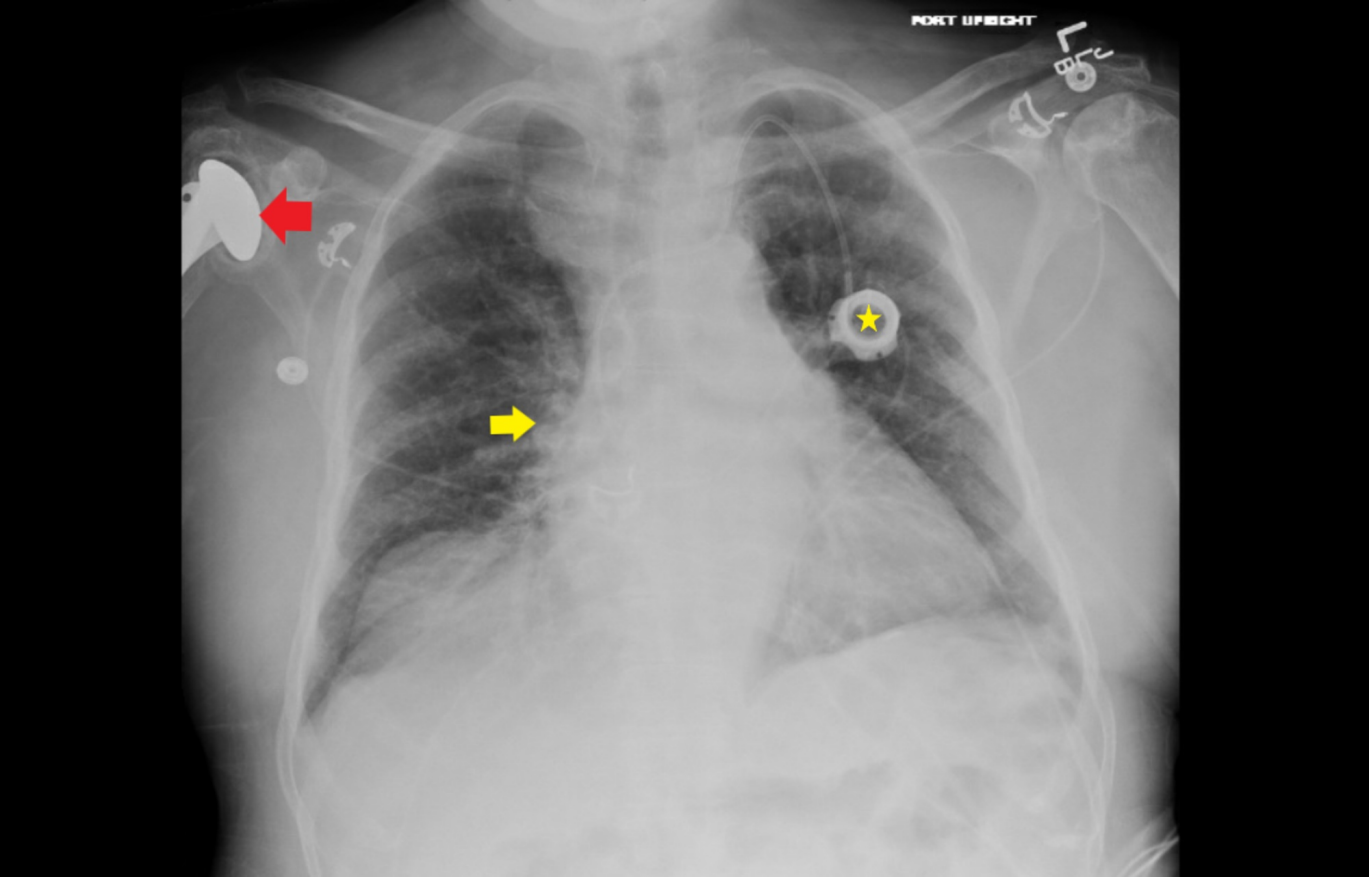
Chest X-ray: bilateral pulmonary hilar congestion (yellow arrow) and bilateral interstitial opacities; left chest wall port (yellow star) with catheter tip at the superior cavoatrial junction; left humeral head osteonecrosis with partially visualized right humeral prosthesis (red arrow)

**Figure 2:**
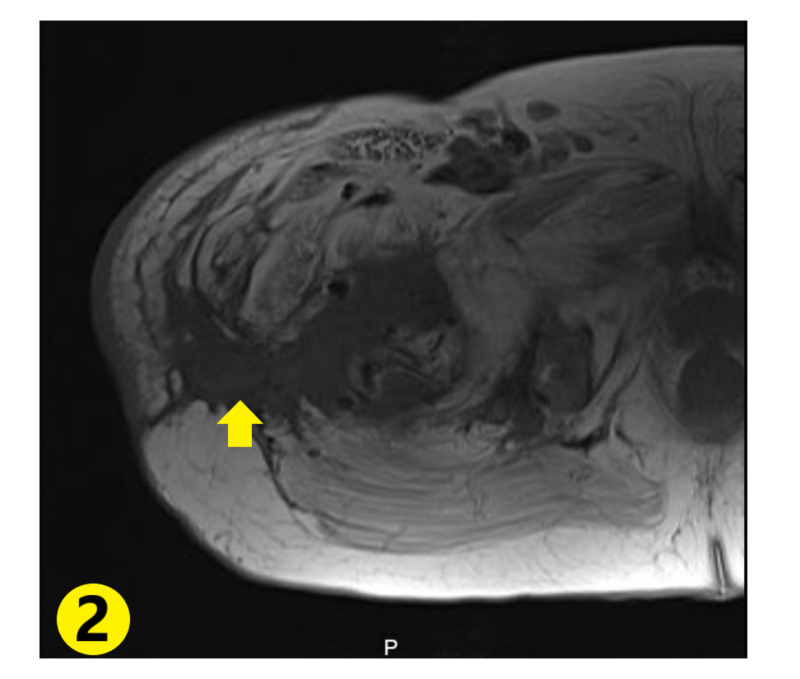
Abscess with surrounding soft tissue enhancement extending from the bone to the outer muscular compartments (yellow arrow)

**Figure 3:**
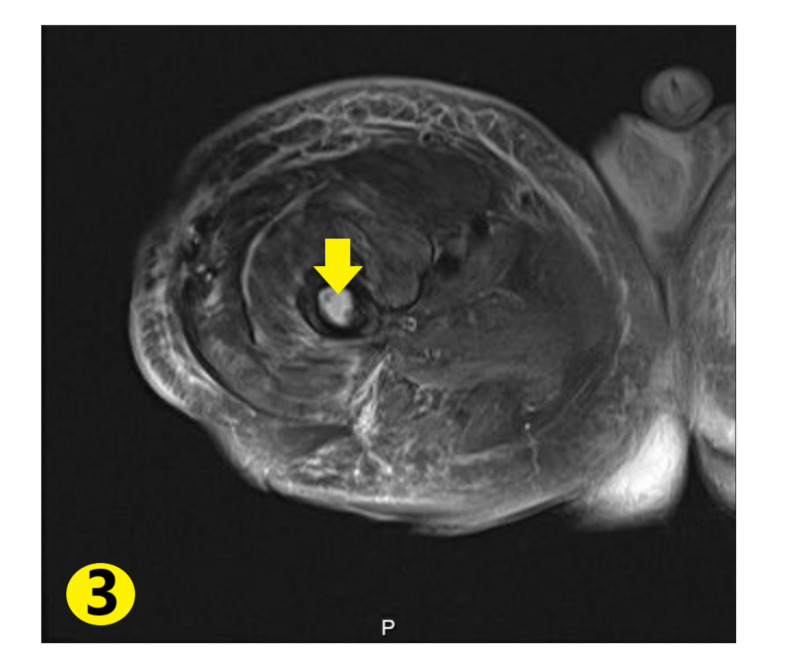
Intramedullary fluid signal enhancement in the femur (yellow arrow) with surrounding soft tissue enhancement

Autoimmune workup showed erythrocyte sedimentation rate (ESR) was mildly elevated to 24 mm/hr, C-reactive protein (CRP) was borderline elevated, creatine kinase (CK) and myoglobin were within normal limits. Autoimmune antibodies returned negative. Chest X-ray revealed pulmonary vascular congestion (Figure 1). MRI of the right hip revealed lateral hip having peripherally enhancing soft tissue fluid collection extending into the right acetabular fossa and into the proximal femur, measuring approximately 5.3 x 14 x 20 cm enlarging soft tissue fluid collection (Figures 2-3). These areas had low signal intensity on T1-weighted imaging and high signal intensity on fluid-sensitive sequences. These signals extended along the lateral aspect of the right hip with intraosseous femoral extension, alongside evidence of femoral and acetabular subacute osteomyelitis. These radiological findings were consistent with Brodie’s abscess.

Orthopedic surgery was consulted for abscess drainage. During the operation, a large amount of pus was found within the thigh muscles, which was traced back and was seen extending from the bone. The abscess was debrided and irrigated, followed by antibiotic bead placement. Clindamycin was added to the antibiotic regimen. After the procedure, his leukocytosis improved drastically.

Wound samples from the abscess were sent for culture. The post-operative course was complicated by the re-accumulation of fluid. Repeat imaging revealed hematoma formation. It was managed conservatively with serial imaging, showing a receding size of the hematoma. Intra-operative culture report revealed the growth of *Enterobacter cloacae* and *Citrobacter koseri*. Antibiotics were changed according to the cultures. The patient made a slow recovery thereafter and was discharged to a rehabilitation facility for recuperation. 

## Discussion

Brodie’s abscess was first acknowledged as a separate entity from acute osteomyelitis in 1823 [3,4]. It was defined as a rare form of subacute or chronic osteomyelitis, characterized by a lack of systemic signs [7]. The average age of involvement is 19.5 years with a male predominance with a male to female ratio of 3:2 [4]. The most common presenting complaint is pain, seen in up to 80% of the cases, followed inconsistently by limping (60%-50%), and swelling (50%-100%) [3]. Metaphyseal involvement of long bones, with tibia being the most commonly involved bone, is more often found in adolescents and children. However diaphyseal involvement has also been reported in the past and is also the more frequently seen in the adult population [5].

The frequently isolated pathogen is similar to acute osteomyelitis and includes *Staphylococcus aureus* [4]. *Pseudomonas aeruginosa*, *Klebsiella spp.*, and *Salmonella typhi* have been reported in the literature [5]. *Salmonella typhi* has an increased propensity for osteomyelitis in sickle-cell disease patients [8]. In 25% of the cases, no pathogenic organism can be identified. Negative intra-operative cultures should increase suspicion of Brodie’s abscess secondary to fastidious organisms. Increased availability of DNA-based testing may improve diagnostic yield in cases of negative bone cultures [3].

Patients are characteristically asymptomatic in the early phases of the disease. White cell count, ESR, and CRP may be normal in up to 50% of the cases [4]. These markers correlate clinically and rise with clinical symptoms. This is hypothesized to be when the integrity of the sclerotic rim of bone is unable to contain the abscess and the contents reach beyond the confines of the sequestered infection [7]. Our patient presented with negative blood cultures, a normal WBC, and elevation of CRP and ESR [1].

The main modality for diagnosis is through radiographic imaging. Initial X-ray may help delineate sclerotic bone with a central core of radiolucent bone with or without periosteal reaction depending on the proximity of the infection to the cortex or if the infection has expanded to beyond the confines of the medulla [7]. Computed tomography (CT) demonstrates increased sensitivity for detecting the sequestrum within the abscess and delineating any sinus tract linking the abscess within or outside the body compartments [9]. The best modality though thus far is still MRI which shows the characteristic features including the granulation tissue incompletely surrounding the central necrotic debris known as the penumbra sign; this is accompanied by the surrounding periosteal rim enhancement known as the “double line” sign [10].

This helps distinguish late phase Brodie’s abscess from acute osteomyelitis which is non-circumscribed, more diffuse, and lacks the characteristic periosteal reaction due to rapidity of the development process [4]. The penumbra sign, although highly suggestive of Brodie’s abscess, is not pathognomonic. The differential for osseous lesions is broad, including chondroblastoma, osteoid osteoma, simple bone cysts, localized Langerhans cell histiocytosis, and rarely malignant bone tumors [3].

Other modalities, though rarely required, include WBC uptake scan if contrast is contraindicated. Alternatively, for inconclusive studies, single‐photon emission computed tomography (SPECT) has better sensitivity and specificity by giving additional information via the SPECT aspect, yielding a more functionally active component by highlighting disease activity, thereby reducing false positives [6].

Treatment encompasses extensive surgical debridement with thorough removal of all necrotic tissue as well as any granulation tissue which may serve as nidus for recurrence of infection [7]. Intra-operative specimens are vital for determining culprit organisms and for source control. Prolonged antibiotic therapy for used for at least six weeks, unless complications arise. Intra-operative antibiotic bead therapy is frequently instituted, which was done in our case [4].

For lesions greater than 3 cm and for fracture risk assessment tool (FRAX) score predicted risk of >20%, bone grafting is considered as an adjunct therapy to provide support and prevent collapse in face of a large cavity. However, this is subject to complete eradication of infection prior to the introduction of healthy bone [4]. Our approach included applying powdered antibiotics with a bone substitute for the local elucidation of antibiotic therapy in combination with long-term IV therapy for infection control, which can be another viable option in the treatment of Brodie’s abscess [2].

Brodie’s abscess is a unique entity among osteomyelitis described as a “geographic, lytic lesion with moderately well- or well-defined edges and without soft tissue mass, matrix, cortical destruction, or bony enlargement occurring in the long bones” [4]. The characteristic thickened sclerotic margins are due to an intact immune system trying to limit the infection from spreading into the bloodstream. Though this shields the presentation in otherwise healthy patients rendering them asymptomatic. However, there is very little data as to why Brodie’s abscess is also found in immunocompromised patients, despite their lack of ability to limit the infection. To our knowledge, there is only one case of Brodie’s abscess in a sickle-cell disease patient which is a state of relative immunodeficiency and has it’s own unique predisposition to osteomyelitis and array of causative pathogens [9].

Our case stands out because of the predisposition to the development of Brodie’s abscess in a male patient with osteoporosis. This osteoporosis is likely due to chronic, subclinical vaso-occlusive crisis in a sickle cell disease patient, causing microscopic osteonecrosis. This, in turn, puts patients at an increased risk for pathogenic organism seeding besides the increased fracture risk.

Besides the lack of characteristic laboratory findings, muddled presentation due to the vaso-occlusive crisis contributed significantly to the late recognition of the disease process. These patients are mistaken for having recurrent vaso-occlusive crisis or pain seeking behavior due to frequent visits and prolonged stay for these complaints. They complain of minor increase in baseline pain but are tolerable with minimal escalation of pain regimen. The patient was diagnosed after an incessant increase in pain medication with little improvement and poorly localized pain. An MRI revealed the underlying Brodie’s abscess. Lastly, the unusual pathogen profile, infection with *Enterobacter cloacae*, and *Citrobacter koseri* has not been previously reported in literature for Brodie’s abscess to our knowledge.

The patient's thigh pain was initially attributed to vaso-occlusive crisis and the diagnosis of Brodie’s abscess was made only after MRI of lower extremities was obtained for persistent fevers. To our knowledge, only one prior case of Brodie’s abscess masquerading as sickle cell vaso-occlusive crisis has been reported [11].

## Conclusions

Brodie’s abscess should, therefore, be considered in the differential for a patient with sickle-cell disease presenting with vaso-occlusive pain crisis. This case was worth reporting as it highlights the importance of having a low threshold for radiologic investigation for unrelenting pain and functional inability to ambulate in sickle-cell disease patients in apparent vaso-occlusive crisis
